# A Modified Mediterranean Diet Improves Fasting and Postprandial Glucoregulation in Adults with Overweight and Obesity: A Pilot Study

**DOI:** 10.3390/ijerph192215347

**Published:** 2022-11-20

**Authors:** Aristea Gioxari, Maria G. Grammatikopoulou, Christina Katsarou, Demosthenes B. Panagiotakos, Marina Toutouza, Stavros A. Kavouras, Labros S. Sidossis, Maria I. Maraki

**Affiliations:** 1 Department of Nutritional Science and Dietetics, School of Health Science, University of the Peloponnese, Antikalamos, GR24100 Kalamata, Greece; 2 Department of Rheumatology and Clinical Immunology, Faculty of Medicine, School of Health Sciences, University of Thessaly, Biopolis, GR41110 Larissa, Greece; 3 Laboratory of Nutrition & Clinical Dietetics, Department of Nutrition and Dietetics, Harokopio University, 70th Eleftheriou Venizelou Str., GR17676 Athens, Greece; 4 Department of Microbiology and Immunology, Hippokration General Hospital, 2–4 Mesogeion Avenue, GR11527 Athens, Greece; 5 Hydration Science Lab, College of Health Solutions, Arizona State University, Phoenix, AZ 85004, USA; 6 Department of Kinesiology and Health, Rutgers University, New Brunswick, NJ 08854, USA; 7 Department of Nutrition and Dietetics, School of Health Sciences, Hellenic Mediterranean University, GR72300 Sitia, Greece; 8 Section of Sport Medicine and Biology of Exercise, School of Physical Education and Sport Science, National and Kapodistrian University of Athens, 41 Ethnikis Antistaseos Str., GR17237 Athens, Greece

**Keywords:** weight, nutrition, diabetes, oral glucose tolerance test, mixed-meal tolerance test, HOMA-IR, adiponectin–leptin ratio

## Abstract

The ideal lifestyle intervention to battle both obesity and diabetes is currently unknown. The aim of this pilot uncontrolled intervention trial was to assess the effect of a modified Mediterranean diet (MedDiet) on weight loss and glucoregulation among overweight/obese adults. Eleven men and women with overweight/obesity, aged 37 ± 12 years, participated in a free-living intervention until 10% weight loss was achieved. Participants followed an individualized MedDiet high in monounsaturated fat and protein with decreased carbohydrate and saturated fat contents. Physical activity and dietary intake were monitored with pedometers and food records, respectively. Upon weight loss achievement, anthropometric measurements, blood metabolic profiles and individual responses to oral glucose and mixed-meal tests were evaluated pre- and post-intervention. The results showed significant ameliorations in body fat, waist circumference and leptin levels (*p* < 0.01), with concomitant increases in adiponectin–leptin ratios (*p* < 0.001). Glucoregulation was significantly improved according to glucose and insulin responses, homeostatic model assessment of insulin resistance indices and postprandial insulin sensitivity indices (*p* < 0.05). In conclusion, the modified Mediterranean diet may induce significant improvements in body composition, adipocytokine profile and glucose metabolism in overweight/obese individuals. Notably, ameliorated glycemia and increased insulin sensitivity may be retained even at postprandial level, irrespective of the meal consumed.

## 1. Introduction

The alarming increase in rates of overweight and obesity worldwide [1] and the ongoing threat of obesity-related metabolic disorders require immediate and targeted intervention [2]. Lifestyle modification is considered the first therapeutic option, but the nature of effective lifestyle interventions is still under investigation [3,4].

Overweight is often associated with reduced insulin sensitivity, leading to the progressive development of prediabetes and diabetes, which further contribute to cardiovascular (CV) risk and all-cause mortality [5,6,7]. Therefore, the primary focus of a lifestyle intervention is reduction in body weight (BW) and improvement in insulin sensitivity [8,9]. Within this context, a plethora of clinical trials have been conducted, with the majority investigating the effectiveness of low-fat, reduced-energy diets [10,11]. Nevertheless, among Mediterranean populations for whom olive oil is an integral part of their diets, compliance to such fat-limited diets might be poor. In fact, a growing body of evidence indicates that adherence to the Mediterranean diet (MedDiet) is associated with improved glucoregulation, blood lipid levels and overall metabolic profile, presumably due to the types of fat sources it includes, these being predominantly monounsaturated fatty acids (MUFAs) and limited amounts of saturated fatty acids (SFAs) [12,13]. However, the MedDiet per se does not lead to substantial weight loss, unless it is energy-restricted [14,15] or modified [16].

For many years, moderate-protein diets (~25% of total energy intake) were associated with increased CV hazard; however, recent data suggest that such diets induce weight loss via a triple mechanism: (i) by enhancing satiety, (ii) by maintaining lean body mass and resting energy expenditure (REE) during weight loss and (iii) via the increased thermic effect of protein [3,17]. It is suggested that the adoption of hypocaloric, low-carbohydrate, moderate-protein diets ameliorates body composition and metabolic profile in overweight individuals, while the previous concerns regarding CV risk have been allayed [3,10,18,19,20]. In fact, both the American Diabetes Association and Diabetes UK have endorsed low-carbohydrate diets as effective therapeutic options for diabetes [8,9].

Therefore, a synthesis of the evidence presented in the literature indicates that a high-MUFA, moderate-protein, low-SFA MedDiet could be effective in achieving weight loss and improving glucoregulation to battle both overweight and reduced insulin sensitivity. The aim of the present pilot prospective study was twofold: (a) to investigate whether a modified non-energy-restricted MedDiet could induce 10% weight loss in overweight/obese sedentary adults and (b) to assess fasting and postprandial insulin sensitivity as well satiety hormones.

## 2. Materials and Methods

### 2.1. Study Design and Protocol Registry

The present pilot study followed an uncontrolled trial design and is reported according to the Standard Protocol Items: Recommendations for Interventional Trials (SPIRIT) statement [21]. The study protocol was registered with the Open Science Foundation (OSF, https://osf.io/vdz4r/?view_only=507968854663433a87f629d2f4f5dee7, accessed on 19 November 2022).

### 2.2. Participants

Metabolically healthy men and women with overweight or obesity were enrolled in the study. All participants were adults (≥18 years) with a body mass index (BMI) equal to or greater than 25 kg/m^2^. The exclusion criteria were: (a) acute or chronic illness, including cardiometabolic disorders (hypertension, diabetes, dyslipidemia, cardiovascular diseases etc.), (b) contraindication to diet, (c) use of medication or dietary supplements known to affect the investigated variables, (d) adherence to special/low-energy diets during the last 6 months, (e) having experienced weight fluctuations of more than 2 kg at any time during the last 2 months, (f) unwillingness to reduce body weight, (g) being pregnant or lactating and (h) being physically active (defined as participation in regular exercise more than once weekly).

### 2.3. Ethics

The study was approved by the Ethics Committee of Harokopio University and was performed in accordance with the ethical standards laid down in the 1964 Declaration of Helsinki and its later amendments. All subjects provided informed consent prior to participation.

### 2.4. Dietary Intervnetion

**Figure 1:**
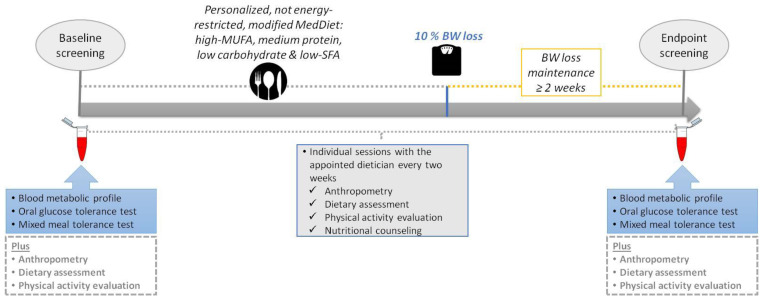
Study design.BW, body weight; MUFA, monounsaturated fatty acid; SFA, saturated fatty acid.

The subjects participated in a free-living dietary intervention, aiming to induce a moderate weight loss, namely, 10% of their initial BW. For each participant, an experienced dietician was appointed who supervised him/her throughout the study. Individual sessions with the dietician were conducted every other week, until a 10% BW loss was achieved and maintained for at least two weeks. At each session, a personalized dietary plan was provided according to the participant’s needs, habits and preferences. Figure 1 details the overall study design.

**Figure 2:**
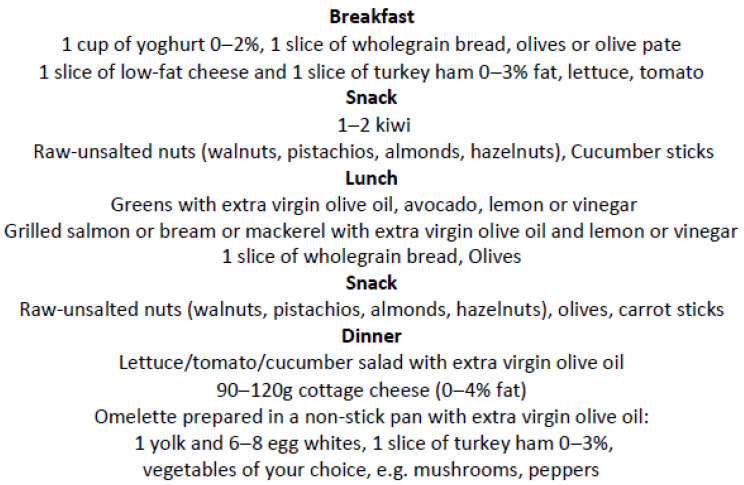
A sample of the modified MedDiet plan.

The dietary intervention was based on the MedDiet with a few modifications in order to achieve a low carbohydrate and low SFA intake paired with moderate protein and total fat consumption [22]. In further detail, consumption of extra virgin olive oil, fish, raw and unsalted nuts (hazelnuts, almonds, walnuts and pistachios), and non-starchy vegetables was unlimited. With regard to intake of starchy foods, milk/yogurt and fruits, consumption was limited to 1–2 food exchanges per each food group, using the US food exchange list for meal planning [23]. In addition, participants were advised to avoid consuming fried foods, ultra-processed foods and fast food, as well as to reduce fat intake from red meat, poultry and dairy products. All participants were encouraged to prepare meals by themselves. Although the design involved ad libitum energy intake, previous studies have shown that a decrease in carbohydrate consumption results in an involuntary reduction in energy intake, possibly due to restricted food choices and diminished appetite [24]. An example of the modified MedDiet plan is shown in Figure 2. Individualized recommendations aiming at increasing daily physical activity were also provided, such as using the stairs instead of the elevator, parking at a distance from work and walking, strolling, etc. 

When a 10% reduction in BW was recorded, a weight-maintenance diet was prescribed. More specifically, 45–50% of energy intake was to be derived from carbohydrates, 30–35% from fats and 20–25% was to be adjusted, if necessary, so that BW fluctuations did not exceed 0.3 kg for at least two weeks. During this weight-maintenance period, participants were instructed to adhere to their typical physical activity patterns (sedentary lifestyle; <5000 steps per day) [25].

### 2.5. Assessments

#### 2.5.1. Anthropometry

Stature and BW were measured with a calibrated stadiometer (Holtain, Crymych, UK) and a calibrated medical beam scale (SECA scale; SECA, Hamburg, Germany) to the nearest 0.1 cm and 0.1 kg, respectively. Waist circumference was measured with a common measuring anelastic tape in a horizontal plane, midway between the inferior margin of the ribs and the top of the iliac crest to the nearest millimeter. Fat mass was determined via dual energy X-ray absorptiometry (model DPX-MD; Lunar, Madison, WI, USA), using software version 4.6 and a 15 min scanning time. Resting energy expenditure (REE), expressed as MJ/d and kJ/kg/h, and resting respiratory quotient (RQ) were measured via indirect calorimetry with a ventilated hood system (Sensormedics, Vmax229, Yorba Linda, CA, USA) after the subjects had been in resting position for approximately 30 min. Anthropometrics, body composition and indirect calorimetry were assessed prior to and after the intervention.

#### 2.5.2. Dietary and Physical Activity Assessments

Dietary intake was assessed with food records that were collected throughout the trial. At each individual session with the appointed dietician (every other week), food diaries were obtained and consisted of at least 2 weekdays and 1 weekend day. Unexpected phone calls were also made to receive 24 h dietary recalls. Nutritionist PRO dietary software, version 2.2 (Axxya Systems, FirstDataBank, San Bruno, CA, USA) was used to calculate daily intakes of energy and macronutrients, i.e., proteins, carbohydrates, fat, SFAs, MUFAs, polyunsaturated fatty acids (PUFAs) and cholesterol. In the case of foods that were not included in the dietary analysis software in advance, the nutrition labels placed on the food packaging were recorded and registered in the software package, in order to estimate the intake of macronutrients. Physical activity was evaluated with pedometers, and the output was expressed as the number of steps per day. Treatment adherence was assessed frequently during the trial (every 1–2 weeks), including dietary intake (food diaries and 24 h recalls) and physical activity levels (pedometers).

#### 2.5.3. Oral Tolerance Tests

The oral glucose tolerance test (OGTT) and the mixed-meal tolerance test (MMTT) were performed as has been previously described [22]. The mixed meal comprised a milkshake made of whipped cream, vanilla ice cream and syrup, containing 76 g of carbohydrate, 15 g of protein, 86 g of fat and 4.78 MJ. Both oral tests were performed after overnight fasting (with a week interval) at two time points, i.e., at baseline and after the dietary intervention (Figure 1). All post-intervention measurements were performed at least two weeks after weight stability (determined as ±0.3 kg change in BW) in order to avoid the acute effects of energy imbalance [26,27].

#### 2.5.4. Blood Sampling and Analyses

Blood samples were drawn from each subject before and during the oral tolerance test. Samples were collected into non-heparinized serum tubes (Sarstedt, Leicester, UK), allowed to clot at room temperature (20 °C), spun in a centrifuge (3000 rpm, 10 min, 4 °C), aliquoted and frozen immediately at −80 °C until analysis. For plasma analyses, blood samples were collected into precooled potassium-EDTA Monovettes (Sarstedt, Leicester, UK), immediately centrifuged (3000 rpm, 10 min, 4 °C), aliquoted and frozen at −80 °C until analysis.

Plasma glucose (GLU), triacylglycerol (TAG), total cholesterol (T-CHOL) and high-density lipoprotein (HDL) levels were determined by the enzymatic colorimetric method, using commercially available kits (Alfa Wassermann Diagnostics, Woerden, The Netherlands) on an automated analyzer (ACE Schiapparelli Biosystems, Fairfield, NI, USA). Serum insulin was assessed with an immunoenzymetric fluorescent method, using a commercially available kit (ST AIA-PACK IRI, Tosoh Medics, San Francisco, CA, USA) on an automated analyzer (Tosoh AIA 600II, Tosoh Medics, Inc., San Francisco, CA, USA). Serum hormones and adiponectin–leptin concentrations were both assessed with commercially available enzyme-linked immunosorbent assays (BioVendor Research and Diagnostic, Brno, Czech Republic). The same batch was used for all the analyses. 

#### 2.5.5. Calculations

Whole-body insulin sensitivity was evaluated with the homeostatic model assessment of insulin resistance (HOMA-IR) index in the fasting state [28], calculated as: “fasting serum insulin (μU/mL) × plasma fasting glucose (mmol/L)/22.5”, as well as with the Matsuda Insulin Sensitivity Index (ISI) in the postprandial state [29,30], calculated as: “10,000/square root of [fasting plasma glucose (mg/dL) × fasting serum insulin (μU/mL) × mean postprandial plasma glucose (mg/dL) × mean postprandial serum insulin (μU/mL)]”. Additionally, postprandial responses of blood glucose and insulin were assessed as the total (AUC) and incremental (iAUC) areas under the concentration-versus-time curves, using the trapezoidal rule. Fasting low-density lipoprotein (LDL) was calculated with the Friedewald equation [31]. The ratio of adiponectin to leptin was calculated as a CV risk index [32].

### 2.6. Outcomes of Interest

The primary outcomes included BW loss (10% of initial BW) and changes (Δ) in waist circumference and body fat (as a % of BW), HOMA-IR and adiponectin–leptin ratios. The secondary outcomes involved changes (Δ) in fasting GLU and INS concentrations and REE, HDL, LDL and total cholesterol levels.

### 2.7. Dropouts and Missing Data

From the enrolment until the analyses, none of the participants dropped out or was lost to follow-up. In parallel, no missing values were apparent, as all participants provided results for all the assessments and analyses.

### 2.8. Statistical Analyses

The normality of distribution of continuous variables was assessed using the Kolmogorov–Smirnov test and percentile plots. Pre- and post-intervention postprandial concentrations were not normally distributed and were logarithmically transformed in order to be compared using repeated measures analysis of variance (ANOVA) followed by posthoc tests adjusted for multiple comparisons (Bonferroni’s test). Means for dietary and physical activity data, fasting variables, AUCs, iAUCs and peak postprandial responses were compared using Student’s paired *t*-tests if the data were normally distributed or Wilcoxon’s signed rank tests if the data were not normally distributed. To investigate whether type of meal (OGTT or MMTT) affected the intervention effects on postprandial responses, additional repeated measures ANOVA with type of meal as the between-subject factor was performed. Continuous data are presented as means ± standard deviations (SDs) or medians (1st quartile (Q1), 3rd quartile (Q3)) for normally or not normally distributed variables, respectively. Dichotomous variables are presented as counts. Statistical significance was set at the 5% level (*p*-value < 0.05). All hypotheses tested were two-tailed. Statistical analysis was performed using PASW Statistics 21.0 (SPSS Inc., Hong Kong, China).

## 3. Results

### 3.1. Participants

Eleven adults with overweight or obesity, namely, 8 men and 3 premenopausal women, with a mean BMI of 34.5 (30.8, 35.2) kg/m^2^ and an age of 37 ± 12 years, met the criteria and participated in the study. Their nationality was Greek, and they were all residents of Athens (Greece). All participants completed the trial and were included in the final analysis.

### 3.2. Anthropometry, Body Composition and REE Measurements

Alterations in anthropometric indices at baseline and after the dietary intervention are shown in Table 1. Participants lost 10.4 ± 1.8 kg of their initial BW (approximately 10%; *p* < 0.001) after 13 ± 5 weeks of following the modified MedDiet. Compared to the baseline, waist circumference, body fat (% BW) and fat mass were significantly reduced post-intervention (*p* < 0.05). Although weight loss induced subsequent reductions in REE (*p* = 0.007), this difference was diminished and was no longer significant when adjusted for body mass (i.e., when REE was expressed as energy per kg of body weight). No significant changes were recorded in the resting RQs of the subjects.

### 3.3. Dietary Intake and Physical Activity

The participants’ dietary intakes and physical activity levels at baseline and during the dietary intervention (every 1–2 weeks) are presented in Table 2. Compared to the baseline, participants following the modified MedDiet significantly reduced their daily intake of energy (*p* < 0.01), carbohydrates (*p* < 0.01) and SFAs (*p* = 0.005), with a subsequent increase in the proportion of energy derivedfrom protein and MUFAs (*p* < 0.01). The ratio of unsaturated to saturated fatty acids increased compared to their pre-intervention intakes (*p* < 0.01). No significant differences were recorded concerning cholesterol intake. Physical activity, expressed as number of daily steps, was increased by approximately 23% during the intervention, but did not achieve statistical significance (*p* = 0.070).

### 3.4. Effects on Fasting Metabolism

Metabolic markers in the fasting state were measured on the morning of OGTT and MMTT, at baseline and post-intervention (Table 3). Fasting insulin concentrations and basal insulin resistance (HOMA-IR index) were significantly reduced after the MedDiet intervention (*p* = 0.001). Concentrations of fasting glucose were also improved (*p* = 0.007). Implementation of the modified MedDiet did not induce significant differences in fasting plasma TAG, T-CHOL, HDL or LDL. On the other hand, circulating leptin levels were significantly limited (*p* = 0.005) and a tendency towards an increase in adiponectin concentrations was recorded, resulting in elevated adiponectin–leptin ratios(*p* = 0.001).

### 3.5. Effects on Postprandial Metabolism

**Figure 3:**
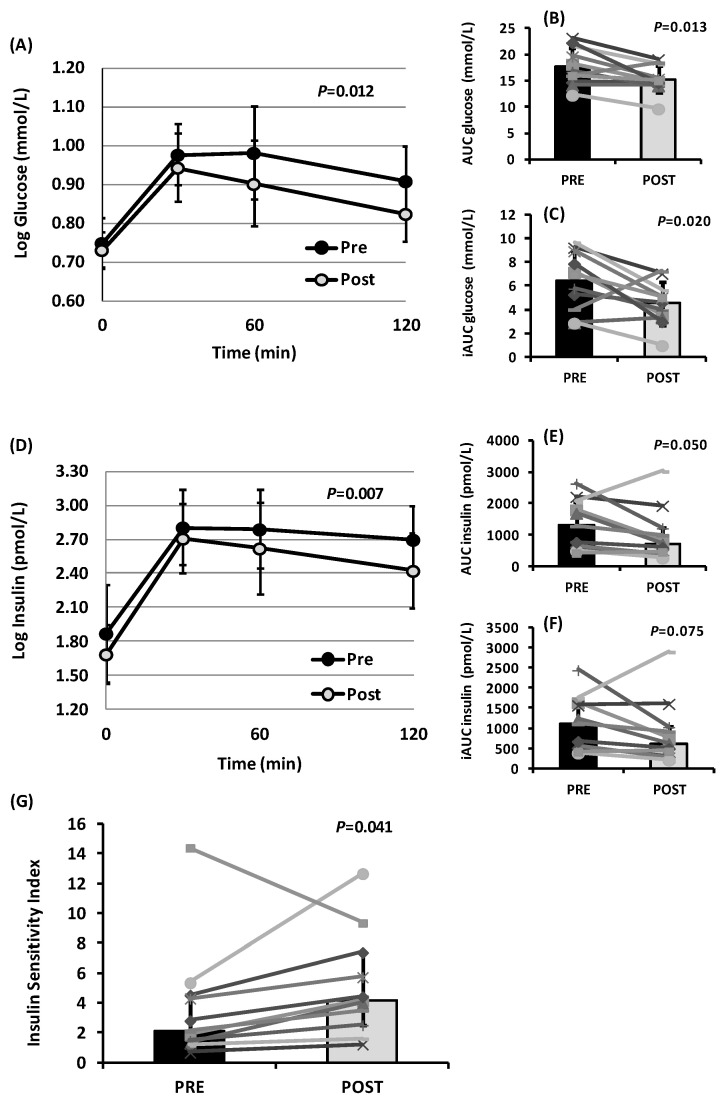
Oral glucose tolerance test (OGTT) in overweight and obese participants before (pre) and after (post) the dietary intervention with the modified Mediterranean diet (n = 11). (**A**) Postprandial plasma glucose. (**B**) Total areas under the curves (AUCs) for glucose. (**C**) Incremental areas under the concentration-versus-time curves (above fasting values, iAUCs) for glucose. (**D**) Postprandial serum insulin. (**E**) AUCs for insulin. (**F**) iAUCs for insulin. (**G**) Postprandial ISI. *p*-values were derived from paired *t*-tests or Wilcoxon rank tests or ANOVA for repeated measures. Statistical significance was set at the 5% level. ANOVA, analysis of variance; ISI, insulin sensitivity index.

Figure 3 depicts postprandial responses to OGTT prior to and post-intervention. Upon achieving 10% weight loss with the modified MedDiet, postprandial plasma glucose concentrations were significantly lower compared to pre-intervention (*p* = 0.012; Figure 3A). Posthoc Bonferroni comparisons revealed that glucose concentrations 1h and 2h after glucose ingestion tended to fall, but without reaching statistical significance. Nevertheless, postprandial total and incremental glucose responses (AUCs and iAUCs) to glucose ingestion were approximately 14% and 30% lower at the study endpoint compared to the baseline, respectively (*p* = 0.013 and *p* = 0.020, respectively; Figure 3B,C). Additionally, postprandial serum insulin concentrations during the OGTT were significantly lower after the MedDiet intervention compared to the baseline (*p* = 0.007; Figure 3D). After dietary intervention, AUCs and iAUCs for insulin were lower in 9 out of 11 participants; hence, the median AUCs and iAUCs tended to be lower by approximately 44% (*p* = 0.050 and *p* = 0.075, respectively; Figure 3E,F). The majority of the subjects (10 out of 11) demonstrated an increase in the Matsuda ISI during the OGTT, resulting in approximately 98% improvement in postprandial insulin sensitivity post-intervention (*p* = 0.041; Figure 3G).

**Figure 4:**
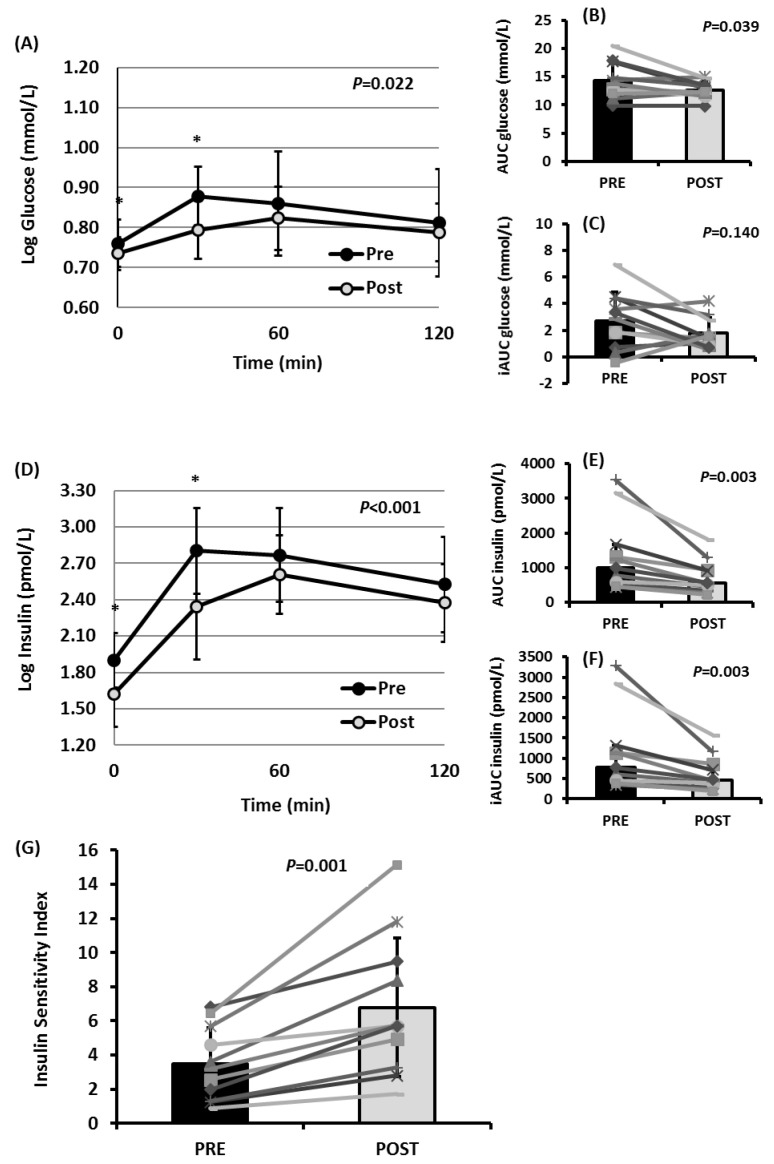
Mixed-meal tolerance test (MMTT) in overweight and obese participants before (pre) and after (post) the dietary intervention with the modified Mediterranean diet (n = 11). (**A**) Postprandial plasma glucose. (**B**) Total areas under the curve (AUCs) for glucose. (**C**) Incremental areas under the concentration-versus-time curves (above fasting values, iAUCs) for glucose. (**D**) Postprandial serum insulin. (**E**) AUCs for insulin. (**F**) iAUCs for insulin. (**G**) Postprandial ISI. *p*-values were derived from paired *t*-tests or Wilcoxon rank tests or ANOVA for repeated measures. Statistical significance was set at the 5% level. ANOVA, analysis of variance; ISI, insulin sensitivity index. * *p* < 0.05 according to posthoc Bonferroni comparisons.

Figure 4 depicts postprandial responses to MMTT prior to and post-intervention. During the MMTT, postprandial plasma glucose concentrations were significantly lower at the study endpoint compared to baseline (*p* = 0.022; Figure 4A). Posthoc Bonferroni comparisons revealed that glucose concentrations before and 1h after meal ingestion were significantly reduced post- compared to pre-intervention (*p* = 0.033 and *p* < 0.001; Figure 4A). Further on, AUCs for glucose were approximately 12% lower, while iAUCs did not significantly change (*p* = 0.039 and *p* = 0.140, respectively; Figure 4B,C). Postprandial serum insulin concentrations during the MMTT were significantly lower after the dietary intervention compared to the baseline (*p* < 0.001; Figure 4D). Posthoc Bonferroni comparisons revealed that insulin concentrations before and 1h after meal ingestion were significantly reduced post-intervention compared to the baseline (*p* = 0.001 and *p* = 0.003; Figure 4D). Similarly, AUCs and iAUCs for insulin were approximately 45% and 42% lower, respectively (*p* = 0.003 and *p* = 0.003, respectively; Figure 4E,F). All subjects demonstrated an increase in the Matsuda ISI during the MMTT, resulting in approximately 95% greater postprandial insulin sensitivity at the study endpoint (*p* = 0.001; Figure 4G).

No significant interactions were observed between type of meal tested, i.e., glucose vs. mixed meal (*p*-values for interactions: 0.648, 0.465, 0.296, 0.200, 0.197, 0.236 and 0.170, for postprandial glucose concentrations, AUCs and iAUCs for glucose, postprandial insulin concentrations, AUCs and iAUCs for insulin, and ISI, respectively).

## 4. Discussion

In the present prospective study, overweight/obese individuals followed a personalized, non-energy-restricted dietary plan based on the MedDiet with a few modifications in order to achieve lower carbohydrate and SFA intake paired with moderate protein consumption. The modified MedDiet was adopted until a 10% weight loss was accomplished. The results showed significant reductions in body fat accompanied by improved glucoregulation. In fact, both fasting and postprandial glucose and insulin concentrations were significantly ameliorated, while insulin resistance was significantly decreased. Additionally, circulating leptin and adiponectin–leptin ratios were significantly improved.

The Mediterranean dietary pattern has long been recognized for its benefits with respect to metabolic health, including weight loss and CV risk reduction in overweight/obese individuals [10,33]. Adherence to the MedDiet has been associated with an increased consumption of low-energy-dense foods, but this assumption is still under consideration. According to the PREDIMED program, adults following a non-calorie-restricted MedDiet enriched with extra virgin olive oil or nuts achieved a 50% risk reduction in developing diabetes and cardiovascular disease compared to those adopting a low-fat diet over a 4-year follow-up period [34,35]. Further on, in the meta-analysis by Mancini and co-workers, the MedDiet induced greater long-term (≥12 months) weight loss than a low-fat diet, but had similar outcomes when compared to a low-carbohydrate diet or the American Diabetes Association Diet [14]. It seems that weight loss following a low-carbohydrate is similar to that for a balanced diet [36]. Oliveira and co-workers argued that discrepancies among studies stem from the different sources of protein intake [37]. Different protein types seem to evoke distinct effects on the satiety mechanism and the production of orexigenic hormones, all of which are related to the timing of ingestion and amino acid threshold levels [38,39]. Compared to plant protein, animal protein is richer in branched-chain amino acids and has been associated with significant improvements in insulin secretion and sensitivity and subsequently glucose uptake [37].

It is well-documented that caloric restriction and exercise have additive beneficial effects on glucoregulation and insulin sensitivity [40]. Although the dietary intervention in the present study was not designed to be hypocaloric, the observed carbohydrate restriction by approximately 45% and the increase in protein intake of approximately 55% significantly contributed to a decline in daily energy intake, leading to weight loss. These changes were accompanied by favorable alterations to fat intake, since SFA consumption decreased (≈−11%) and MUFA consumption increased (≈110%)—actions that were both in line with the typical Mediterranean diet regimen [41]. According to a recent crossover trial conducted in patients with type 2 diabetes, adherence to a non-energy-restricted, carbohydrate-reduced, high-protein diet for six weeks was associated with ameliorated glucoregulation (as assessed by fasting and postprandial glucose AUC after the ingestion of a mixed meal) compared to an isocaloric control diet [42]. In the same study, β-cell function improved, including pro-insulin processing and increased subjective satiety [42]. Furthermore, the carbohydrate-reduced, high-protein diet significantly increased postprandial glucagon net AUC and delayed gastric emptying by 15 min. Similarly, in our study, insulin and glucose responses following the two tolerance tests (OGTT and MMTT) significantly improved, with concomitant ameliorations of leptin levels and adiponectin–leptin ratios. These findings indicate that the improved glucoregulation is not restricted to a fasting level but is actually the result of an improved physiological process.

Weight loss and enhancement of insulin action following a non-energy-restricted, high-protein diet has been associated with improvement in blood lipids [22]. Clifton and co-workers showed that short-term, high-protein weight loss diets might have beneficial effects on triacylglycerol and total cholesterol levels in patients with overweight or obesity [18]. Similar results were observed by Parker and co-workers in patients with type 2 diabetes [43]. Nevertheless, in the present study, blood lipid profiles did not change, probably due to the short time span of the dietary intervention (13 ± 5 weeks) and the small sample size. 

Overall, it has been suggested that a moderate increase in protein intake is associated with increased thermic effect, secretion of satiety hormones (such as GIP and GLP-1), a concomitant reduction in the secretion of ghrelin, as well as alternations in the gluconeogenesis process, all of which may induce improvements in glucose homeostasis [39]. Therefore, it appears that improved satiety, weight loss and increased lipid breakdown act synergically to “normalize” glucoregulation after the adoption of moderate-protein diets. In the present intervention, increase in protein intake was accompanied by an increase in MUFA and a decrease in SFA intake. As seen in the OmniHeart trial, diets low in SFAs but rich in either UFAs or protein may improve HDL and triacylglycerol concentrations, both of which are adversely affected by the adoption of a high-carbohydrate diet [44]. Therefore, the improvements in glycemia recorded among overweight/obese patients in the present study could be additive epiphenomena of both the increase in MUFA and protein intake and the decrease in SFA consumption. 

Another important finding of the present study is the recorded reduction in leptin and a trend towards an increase in adiponectin levels after the implementation of the modified MedDiet. Both hormones are highly dependent on fat mass [45]. During energy restriction, circulating leptin decreases, attenuating weight loss and favoring weight regain [46,47]. In addition, energy restriction tends to increase plasma adiponectin concentrations, attenuating insulin resistance, dyslipidemia and atherosclerosis [45]. This results in an elevated adiponectin–leptin ratio, as observed in our study, suggesting a decrease in overall CV and metabolic risk and improved adipose tissue functionality [48]. 

Nonetheless, the present study also has a few limitations. Research on metabolomics has shown that long-term high intake of branched-chain and aromatic amino acids paired with an increased fat intake facilitates the development of metabolic diseases [39,49]. Consequently, careful selection of the sources and amount of protein is crucial in maintaining health. In addition, the present study might be biased due to the lack of a control group; however, it provides important findings concerning diet composition during BW loss and glucoregulation. An additional limitation is the relatively small group of participants and the unequal sex distribution. Nevertheless, the present trial served as a pilot for the design of future studies with greater numbers of participants.

## 5. Conclusions

The results of the present pilot prospective study indicate that a modification of the MedDiet with a moderate increase in protein and MUFA intake paired with a reduction in carbohydrate and SFA consumption may produce significant improvements in body composition and anthropometric, glucoregulation and adipocytokine profiles in overweight/obese adults. The ameliorated glycemia was retained even at postprandial level, indicating increased insulin sensitivity, irrespective of the meal tested.

## Figures and Tables

**Table 1:** Anthropometric, body composition and REE measurements, prior to and post-intervention.

Anthropometric Indices	Pre-Intervention (n = 11)	Post-Intervention (n = 11)	*p*-Value
Participants Women Men	11 8 3	11 8 3	- - -
BW (kg)	103.1 ± 18.3	92.7 ± 17.5	<0.001
BMI (kg/m^2^) BMI < 25 kg/m^2^ 25 ≤ BMI < 29.9 kg/m^2^ BMI ≥ 30 kg/m^2^	34.5 (30.8, 35.2) 0 1 10	30.9 (27.7, 31.5) 1 4 6	0.003 - - -
Waist circumference (cm)	108.1 ± 16.2	99.9 ± 17.3	<0.001
Body fat (% BW)	39.8 ± 7.9	36.5 ± 7.4	<0.001
Fat mass (kg)	40.4(32.1, 42.1)	33.2 (25.6, 36.0)	0.005
REE (MJ/d)	7.40 (6.75, 7.84)	6.73 (6.31, 7.11)	0.007
REE (kJ/kg/h)	2.94 ± 0.17	3.00 ± 0.26	0.223
Resting RQ	0.81 ± 0.06	0.78 ± 0.09	0.203

Dichotomous variables are presented as counts. Continuous data are expressed as means ± standard deviations of the mean (SDs) or medians (Q1, Q3). *p*-values were derived from paired *t*-tests or Wilcoxon rank tests, according to variable distributions. Statistical significance was set at the 5% level. BMI, body mass index; BW, body weight; REE, resting energy expenditure; RQ, respiratory quotient.

**Table 2:** Dietary intake and physical activity prior to and during the weight-loss intervention.

	Indices	Pre-Intervention (n = 11)	During Intervention (n = 11)	*p*-Value
Daily dietary intake:	Energy (EI)(MJ)	10.93 ± 2.94	6.14 ± 1.64	0.001
	Protein (g)	101 ± 34	87 ± 22	0.235
	Carbohydrate (g)	274 ± 78	87 ± 30	<0.001
	Fat (g)	114 (74, 144)	72 (66, 113)	0.050
	SFAs (g)	47 ± 18	23 ± 8	0.005
	Cholesterol (mg)	292 (256, 398)	221 (189, 233)	0.091
	Protein (% EI)	15.5 ± 3.6	24.1 ± 4.2	<0.001
	Carbohydrate (% EI)	42.4 ± 10.0	23.2 ± 5.5	<0.001
	Fat (% EI)	38.6 ± 9.0	52.7 ± 5.9	0.002
	SFAs (% EI)	15.9 ± 3.1	14.2 ± 4.2	0.346
	PUFAs (% EI)	5.3 (4.1, 5.9)	5.7 (4.5, 7.1)	0.328
	MUFAs (% EI)	13.6 ± 6.2	28.6 ± 5.8	<0.001
	UFAs/SFAs	1.18 ± 0.44	2.68 ± 0.98	0.001
Number of steps per day:		4738 ± 2353	5888 ± 2146	0.070

Values are expressed as means ± standard deviations of the mean (SDs) or medians (Q1, Q3). *p*-values were derived from paired *t*-tests or Wilcoxon rank tests, according to variable distributions. Statistical significance was set at the 5% level. EI, energy intake; MUFAs, monounsaturated fatty acids; PUFAs, polyunsaturated fatty acids; SFAs, saturated fatty acids; UFAs, unsaturated fatty acids.

**Table 3:** Fasting variables before and after the intervention.

	Pre-Intervention (n = 11)	Post-Intervention (n = 11)	*p*-Value
Glucose (mmol/L)	5.47 (5.39, 6.58)	5.39 (5.25, 5.81)	0.007
Insulin (pmol/L)	89.1 ± 46.9	50.0 ± 31.2	0.001
HOMA-IR	3.43 ± 2.14	1.78 ± 1.22	0.001
Triacylglycerol (mmol/L)	1.36 ± 0.68	1.18 ± 0.54	0.159
Total cholesterol (mmol/L)	4.87 ± 0.64	4.68 ± 0.61	0.333
HDL (mmol/L)	0.88 (0.73, 1.63)	0.92 (0.73, 1.14)	0.789
LDL (mmol/L)	3.20 ± 0.51	3.15 ± 0.56	0.723
Adiponectin (μg/mL)	5.44 ± 2.45	6.06 ± 2.27	0.055
Leptin (μg/mL)	22.0 (15.8, 28.8)	10.4 (8.0, 15.9)	0.005
Adiponectin–leptin ratio	0.28 ± 0.20	0.63 ± 0.36	0.001

Values are expressed as means± standard deviations of the mean (SDs) or median (Q1, Q3). *p*-values were derived from paired *t*-tests or Wilcoxon rank tests, according to variable distributions. Statistical significance was set at the 5% level. HOMA-IR, homeostasis model assessment of insulin resistance; HDL, high-density lipoprotein; LDL, low-density lipoprotein.

## Data Availability

We carefully documented the data, methods and materials used for the research in the article. We will share anonymized data at the request of other qualified investigators for purposes of replicating procedures and results.
